# Imaging Aβ and tau in early stage Alzheimer’s disease with [^18^F]AV45 and [^18^F]AV1451

**DOI:** 10.1186/s13550-018-0371-y

**Published:** 2018-03-02

**Authors:** Azadeh Firouzian, Alex Whittington, Graham E. Searle, Ivan Koychev, Giovanna Zamboni, Simon Lovestone, Roger N. Gunn, on behalf of the Deep and Frequent Phenotyping study team

**Affiliations:** 1 Imanova Ltd., Burlington Danes Building, Imperial College London, Hammersmith Hospital, Du Cane Road, London, W12 0NN UK; 2 Department of Medicine, Faculty of Medicine, Imperial College London, South Kensington Campus, London, SW7 2AZ UK; 3 Department of Psychiatry, University of Oxford, Warneford Hospital, Oxford, OX3 7JX UK; 4 Nuffield Department of Clinical Neurosciences, University of Oxford, Oxford, OX3 9DU UK; 5 Department of Engineering Science, University of Oxford, Oxford, UK

**Keywords:** Alzheimer’s disease, PET, Amyloid, Tau, Modelling

## Abstract

**Background:**

AD is a progressive neurodegenerative disorder that is associated with the accumulation of two different insoluble protein aggregates, Aβ plaques and hyperphosphorylated tau. This study aimed to investigate the optimal acquisition and quantification of [^18^F]AV45 and [^18^F]AV1451 to image Aβ and tau, respectively, in subjects with AD.

Fifteen subjects with early stage AD underwent a T1-weighted structural MRI and two dynamic PET scans to image Aβ (60 min, [^18^F]AV45) and tau (120 min, [^18^F]AV1451). Both dynamic BP_ND_ and static SUVR outcome measures were calculated and compared for 12 out of 15 subjects who completed 60 min of the Aβ PET scan and at least 110 min of the tau PET scan. The SRTM and reference Logan graphical analysis were applied to the dynamic data to estimate regional BP_ND_ values and SUVR ratios from the static data. Optimal acquisition windows were explored for both the dynamic and static acquisitions. In addition, the spatial correlation between regional Aβ and tau signals was explored.

**Results:**

Both the SRTM and graphical analysis methods showed a good fit to the dynamic data for both Aβ and tau dynamic PET scans. Mean regional BP_ND_ estimates became stable 30 min p.i. for [^18^F]AV45 and 80 min p.i. for [^18^F]AV1451.

Time stability analysis of static SUVR data showed that the outcome measure starts to become stable for scan windows of 30–50 min p.i. for [^18^F]AV45 and 80–100 min p.i. for [^18^F]AV1451. The results from these time windows correlated well with the results from the full dynamic analysis for both tracers (*R*^2^ = 0.74 for [^18^F]AV45 and *R*^2^ = 0.88 for [^18^F]AV1451). There was a high correlation between amyloid uptake estimate using both dynamic analysis methods in thalamus and tau uptake in thalamus, hippocampus and amygdala.

**Conclusions:**

Short static PET scans at appropriate time windows provided SUVR values which were in reasonable agreement with BP_ND_ values calculated from dynamic scans using SRTM and reference Logan. These simplified methods may be appropriate for classification and intervention studies, although caution should be employed when considering interventional studies where blood flow and extraction could change.

## Background

Alzheimer’s disease (AD) is a progressive neurodegenerative disorder that is associated with the accumulation of two different insoluble protein aggregates, amyloid-β (Aβ) plaques and neurofibrillary tangles (NFTs) consisting of hyperphosphorylated tau protein. Evidence suggests that both Aβ and NFTs have known involvement in AD together with other less explored contributors [1]. The pathophysiological process of AD begins years before the clinical symptoms appear [2–4] and this preclinical phase provides an opportunity for therapeutic intervention. Molecular imaging of Aβ and tau could provide important tools for stratifying subjects in this preclinical phase and assessing the impact of novel drug therapies [5, 6].

Positron emission tomography (PET) radioligands are now available to image both insoluble Aβ plaques and tau neurofibrillary tangles relevant for AD [7]. Aβ imaging has been feasible since 2004 [8] following the introduction of [^11^C]PiB and a number of fluorinated Aβ radiotracers (Florbetapir ([^18^F]AV45), [^18^F]Florbetaben, [^18^F]Flutemetamol) that have been approved by European medicines agency (EMA) as well as food and drug agency (FDA). These tracers are being used in clinical trials for stratification and to assess Aβ levels pre and post therapy [6, 9]. More recently, efforts have focused on developing tracers suitable for imaging tau [10].

Initial studies have demonstrated that there are radiotracers that bind to tau and provide signals that are consistent with postmortem data [11–13]. One of the first generation tau tracers that has demonstrated such signals is [^18^F]AV1451 ([^18^F]T807) with evident in vivo differences between healthy controls and AD subjects that generally reflect the expected spatial distribution of tau in AD [14].

The data presented here, involving [^18^F]AV45 and [^18^F]AV1451, were acquired as part of the pilot phase of the UK medical research council-sponsored (MRC) deep and frequent phenotyping study which aims to identify stratification markers and markers of change in the pre-clinical phase of AD subjects. The pilot phase aimed to determine participants’ acceptability of extensive and repeated phenotyping, the practicality of this procedure and to establish an optimal protocol for the main study.

This paper aims to assess the PET imaging data acquired as part of the pilot study, including dynamic Aβ and tau scans, to provide better understanding of the data and working towards designing an efficient protocol for the main study. Different acquisition (dynamic vs static) and analysis procedures will impact on the outcome measures derived and were investigated with this data set. Full kinetic analysis of the dynamic PET scans was performed using quantitative reference tissue approaches including the simplified reference tissue model (SRTM) [15, 16] and reference Logan graphical analysis [17] to derive the non-displaceable binding potential (BP_ND_). Additionally, the simpler static measure of standardized uptake value ratio (SUVR) was obtained from these data at various time windows and compared against the full dynamic quantification to assess outcome measure stability and bias. Finally, the spatial relationship between Aβ and tau was investigated across the subjects that had been scanned.

## Methods

### Subjects

Fifteen subjects were included in the analysis, with a diagnosis of mild AD (with no AD pathophysiological evidence) according to national institute of aging-Alzheimer’s association (NIA-AA) criteria [18–21], aged between 54 and 83 years, with a mini mental state examination (MMSE) score of 21–29 and a modified Hachinski ischemic score (HIS) of less than 4 [22]. All subjects underwent a series of assessments including clinical, cognitive, gait and ophthalmological assessments, as well as molecular markers in cerebrospinal fluid (CSF), blood, urine, PET imaging, magnetic resonance imaging (MRI), magnetoencephalography (MEG) and electroencephalography (EEG). The study was approved by the institutional review board and all subjects signed an informed consent form (ICF).

### Image acquisition

All subjects underwent a 3D T1-weighted structural MRI and two dynamic PET scans to image Aβ and tau on separate days. Acquisitions were conducted in accordance with the international conference on harmonisation (ICH) guideline for good clinical practice (GCP) and the ethical principles that have their origins in the declaration of Helsinki. The institutional review board/ethics committee reviewed and approved the protocol and ICFs as well as any advertising and subject materials before any subjects were enrolled. A written, signed and dated ICF was provided before any protocol was performed.

The MRI scan was performed on a Siemens 3T Tim Trio with a 32-channel phased array head coil. A 1 mm isotropic whole-brain structural 3D T1-weighted MPRAGE [23] was acquired using TI = 880 ms, TR = 2000 ms and FA = 8° with a parallel imaging factor of 2 in 4 m: 54 s. For the PET scans, subjects were positioned in the PET scanner after the insertion of a venous cannula in an antecubital or forearm vein, and a head-fixation device was used to minimize head movement during data acquisition. The PET scans were acquired on two Siemens PET/CT (computed tomography) scanners (Hi-Rez Biograph 6 and Biograph 6 TruePoint with TrueV, Siemens Healthcare, Erlangen, Germany), though for consistency each subject had both of their PET scans on the same scanner. A low-dose CT scan was performed immediately before each PET scan in order to estimate attenuation. Each subject received a single intravenous bolus of [^18^F]AV45 (150 ± 24 MBq) for a 60 min Aβ PET scan and [^18^F]AV1451 (163 ± 10 MBq) for a 120 min tau scan. The dynamic images were reconstructed using a 2D filtered back projection (FBP) algorithm resulting in a 128 × 128 matrix with 2 mm isotropic voxels. Corrections were applied for attenuation, randoms, scatter and radioactive decay.

### Image analysis

Two different classes of image analysis approaches were investigated in this study: static and dynamic. In both approaches, the T1-weighted MRI scan was used to obtain anatomical information for each subject. Each subject’s whole brain was extracted using the Oxford centre for functional MRI of the brain (FMRIB) software library (FSL) [24] brain extraction tool (BET) [25] and the corresponding grey matter probability maps were created using statistical parametric mapping (SPM5) software [26]. A template MRI (ICBM152 [27]) was then nonlinearly warped to subject’s MRI using SPM5 and the resulting deformation was applied to a brain anatomical atlas [28] consisting of 119 brain regions to obtain the brain region boundaries for each subject. This individualized atlas was used at a later stage to derive regional tissue time activity data from the dynamic PET images.

The dynamic PET images (2 × 2 × 2 mm) were initially motion corrected by rigidly registering each frame to a reference frame (13–15 min p.i.) using mutual information and subsequently rigidly transformed into alignment with the MRI and individualized atlas. The chosen reference frame contains both information on delivery and uptake allowing for both early and late frames to be aligned effectively. Finally, to be able to calculate different parameters, the atlas in the individualized Montreal neurological institute (MNI) space is down-sampled to match PET voxel size.

Regional time activity curves (TACs) were generated using the atlas and dynamic PET images. The SRTM and reference Logan graphical analysis with cerebellum grey matter as reference region were applied to the regional TACs to estimate the BP_ND_, used to quantify the amount of tracer binding to the target proteins. Parametric images for both measures were also created by applying the models to each voxel [16].

The SRTM model equation is given by,

1 $$ {C}_{\mathrm{T}}(t)={R}_1{C}_{\mathrm{R}}(t)+\left({k}_2-\frac{R_1{k}_2}{1+{\mathrm{BP}}_{\mathrm{ND}}}\right){C}_{\mathrm{R}}(t)\otimes {e}^{-\frac{-{\mathrm{k}}_2}{\left(1+{\mathrm{BP}}_{\mathrm{ND}}\right)}\ \mathrm{t}} $$

where *C*_T_(*t*) is the activity concentration in the target tissue, *C*_R_(*t*) is the activity in reference tissue, *k*_2_ is the efflux rate constant from target tissue, *R*_1_ is the ratio of the delivery in target region to reference region. *R*_1_, *k*_2_ and BP_ND_ are estimated for each region.

The reference Logan graphical analysis method is given by,

2 $$ \frac{\int_0^{\mathrm{t}}{C}_{\mathrm{T}}(s)\mathrm{ds}}{C_{\mathrm{T}}(t)}=\left(1+{\mathrm{BP}}_{\mathrm{ND}}\right)\ \frac{\int_0^{\mathrm{t}}{C}_{\mathrm{R}}(s)\mathrm{ds}}{C_{\mathrm{T}}(t)}+\operatorname{int}\kern1.75em \mathrm{for}\kern0.5em t>t\ast $$

where BP_ND_ is the binding potential, ‘int’ is the regression intercept and t^*^ is the equilibrium time.

The distribution of estimated regional BP_ND_ values using the two reference tissue analysis methods from tau and Aβ PET scans over all subjects was compared to assess the performance of each method.

For the static analysis, SUVR values (Eq. 3) were calculated using cerebellum grey matter as the reference region. Regional average SUVR values were calculated for 20 min time windows for a range of different start times post injection (p.i.) (between 0 and 60 min for [^18^F]AV45 and between 0 and 120 for [^18^F]AV1451) from static images created by averaging the frames in each time window.

3 $$ \mathrm{SUVR}(t)=\frac{{\mathrm{SUV}}_{\mathrm{Target}}(t)}{{\mathrm{SUV}}_{\mathrm{Reference}}(t)} $$

where SUV_Target_ is the SUV of the target region and SUV_Reference_ is the SUV of the reference region.

Additionally, the time stability of both dynamic (BP_ND_) and static (SUVR) outcome measures and their relationship were investigated. The BP_ND_ values were calculated for several scan durations by reducing the scan duration with steps of 10 min and the SUVR values were calculated for 20 min time windows created by splitting the scan period into 20 min time windows. The relation between these two measures was assessed by performing a regression analysis. Additionally, the correlation between regional Aβ and tau signals was explored.

All the above mentioned analysis was performed using molecular imaging and kinetic analysis toolbox (MIAKAT™, version 4.0.2, http://www.miakat.org/MIAKAT2/index.html).

## Results

All subjects successfully completed the 60 min Aβ PET scans and 12 out of 15 completed at least 110 min of the tau PET scan—these 12 subjects were included in the analysis that is presented here. The included subjects had an average age of 71.4 ± 10.1, modified HIS score of 0.8 ± 1.0, mild cognitive impairment with MMSE score of 24.3 ± 2.1 and Alzheimer’s disease assessment scale-cognitive (ADAS-Cog) score of 15.5 ± 7.1. Mean symptom duration was 3.5 ± 2.1 years and mean time since the initial diagnosis was 1.3 ± 2.1 years which was confirmed as an inclusion criteria at the time of the PET scan.

### Dynamic analysis

**Fig. 1:**
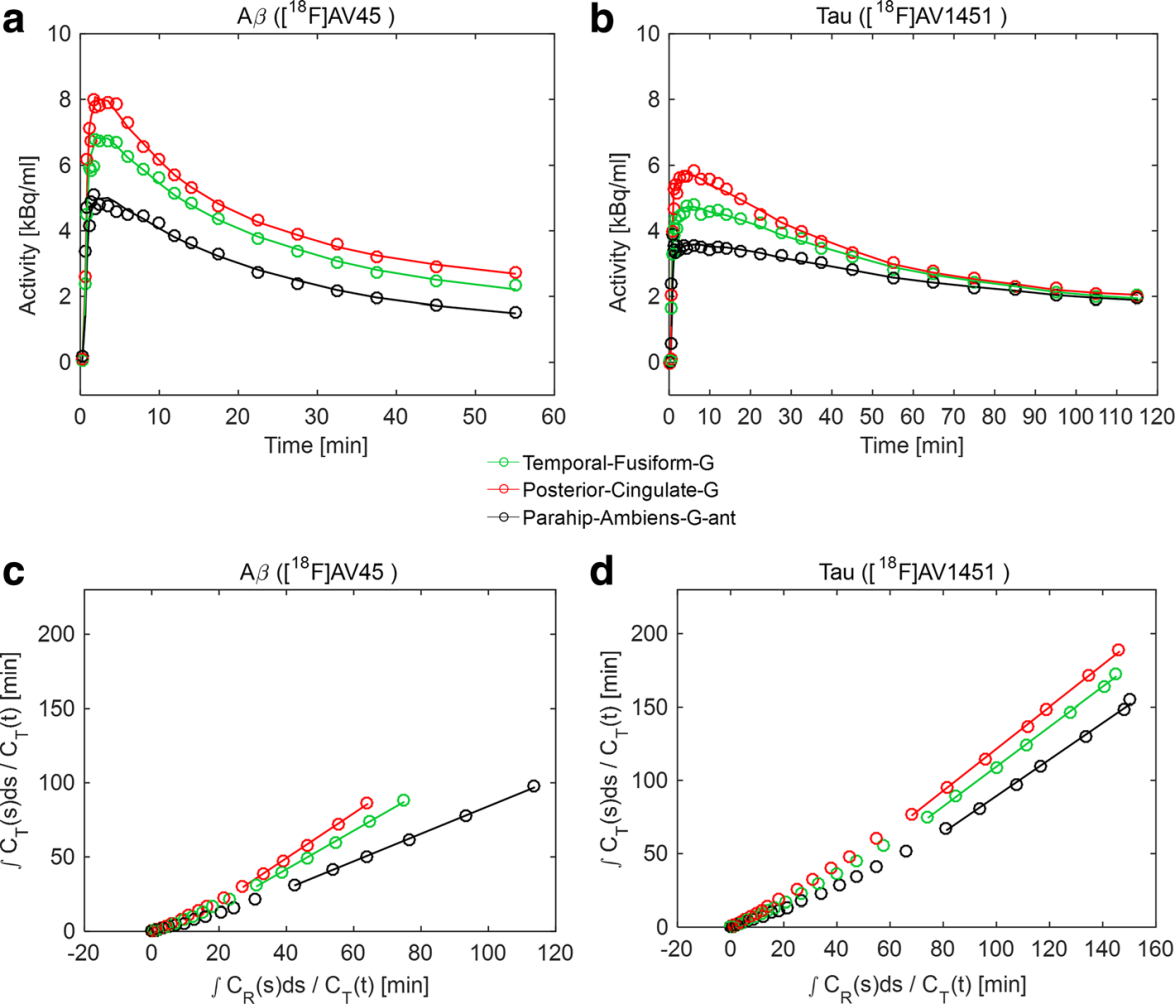
SRTM (**a**, **b**) and reference Logan graphical analysis model (**c**, **d**) fit (solid line) to TACs (circles) of three example ROIs with different activity levels for Aβ (**a**, **c**; t* = 20) and tau (**b**, **d**; t* = 50) dynamic PET scans

TACs and SRTM (Eq. 1) kinetic fits were successfully generated for all 119 atlas regions of interest (ROIs) for both Aβ and tau dynamic PET scans for all subjects. The SRTM with cerebellum grey as reference region was fitted to the TACs for each region (Fig. 1a, b) and the three parameters (R_1_, k_2_ and BP_ND_) were estimated per region. The TACs show that the tau signal from [^18^F]AV1451 has slower kinetics and less regional distinction in comparison to the Aβ signal from [^18^F]AV45. Similarly, reference Logan graphical analysis (Eq. 2) with cerebellum grey as reference tissue was fitted to the TACs (Fig. 1c, d) and the two parameters (BP_ND_ and int) were estimated per atlas region for each subject. Both models showed a good fit to the data for both Aβ and tau dynamic PET scans.

**Fig. 2:**
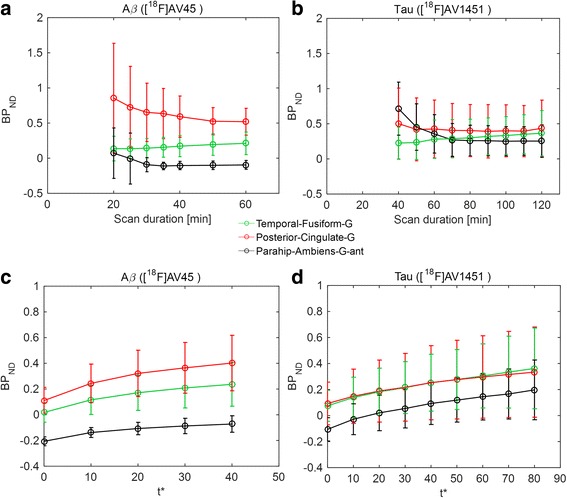
Estimated mean (the circles) and variation (vertical bars) of regional BP_ND_ values using SRTM (**a**, **b**) and reference Logan graphical analysis (**c**, **d**) with cerebellum grey as reference region for three example regions over all subjects for Aβ (**a**, **c**) and tau (**b**, **d**) dynamic PET scans. t* indicates the starting time of reference Logan graphical analysis

**Fig. 3:**
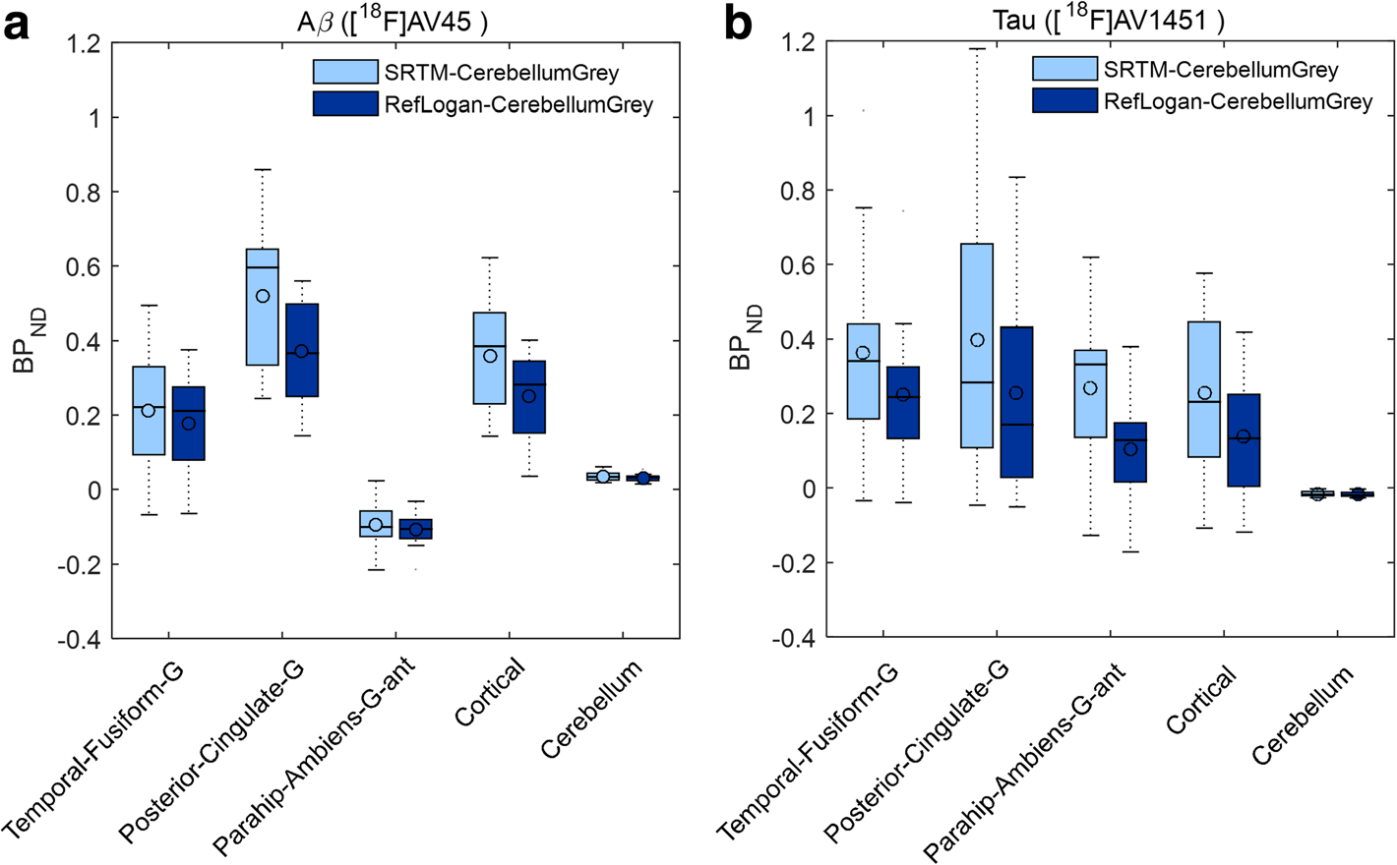
Estimated regional BP_ND_ values using SRTM and reference Logan graphical analysis with cerebellum grey as reference region across all subjects for selected regions for Aβ (**a**) and tau (**b**) dynamic PET scans. The BP_ND_ values estimated by reference Logan graphical analysis are for optimum t* values (t* = 20 for Aβ and t* = 50 for tau)

To investigate the approximate time at which regional BP_ND_ values stabilize, the mean regional BP_ND_ values derived using SRTM averaged over all subjects was plotted against scan duration for both Aβ and tau dynamic PET scans (Fig. 2a, b). Mean regional BP_ND_ estimates become stable after 30 min p.i. for [^18^F]AV45 and after 80 min p.i. for [^18^F]AV1451 for all regions. Similar analysis was performed to the same PET scans using reference Logan graphical analysis model with cerebellum grey as reference region and mean regional BP_ND_ values were estimated for all subjects using a range of starting time points (t*) for the regression analysis (Fig. 2c, d). For t* values of over 30 min, the BP_ND_ values are stable for Aβ scans, whereas stability occurs at t* greater than 70 min for tau. The estimated regional BP_ND_ values using the two dynamic models were compared over all subjects for Aβ and tau dynamic PET scans (Fig. 3). Similar regional BP_ND_ values were estimated by both dynamic models but overall, the regional BP_ND_ values estimated by SRTM were higher on average with larger variation across subjects.

### Static analysis

**Fig. 4:**
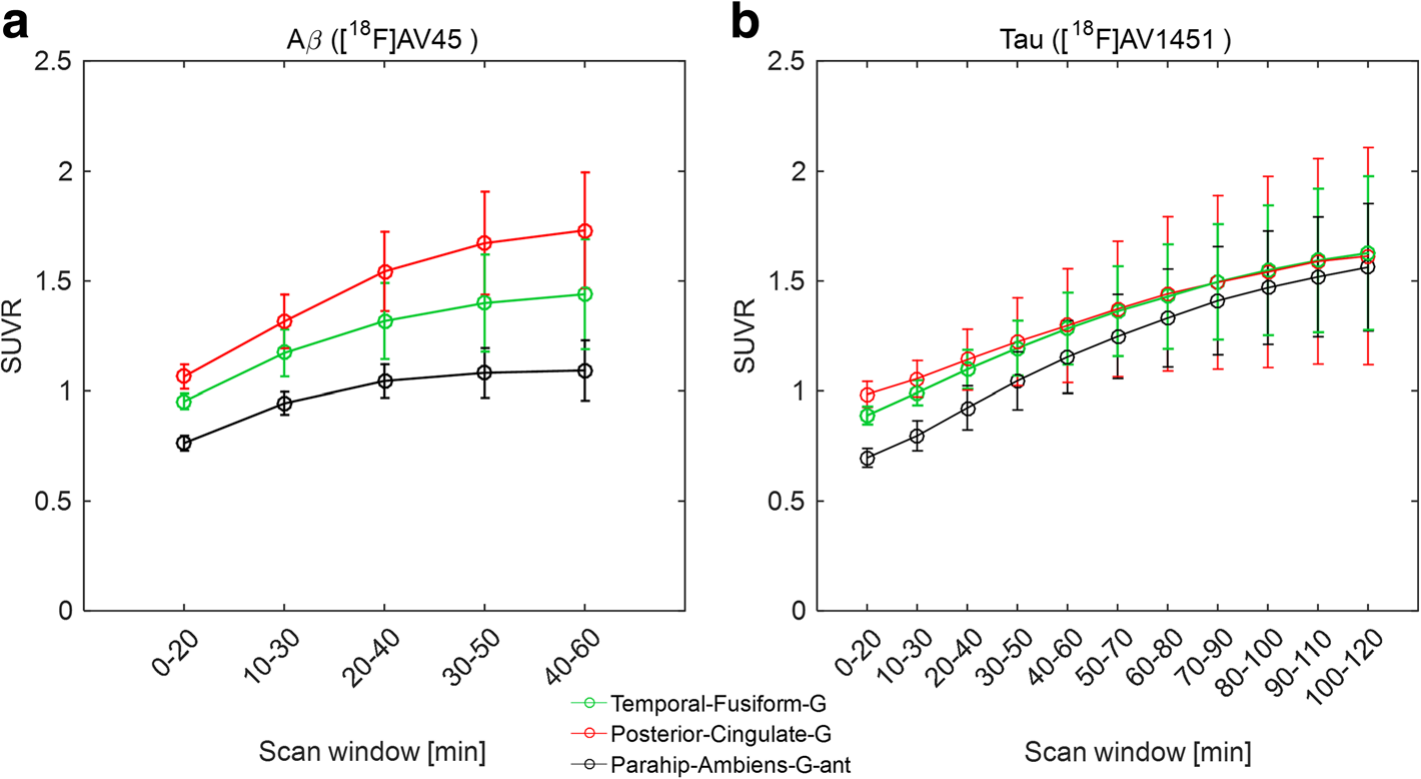
Time stability of mean SUVR values over all subjects with cerebellum grey as reference region for 20 min time windows over the whole scan duration for Aβ (**a**) and tau (**b**) scans for three example regions

The static analysis involved the calculation of regional SUVR values for 20 min scan windows spanning the full scan duration using Eq. 3 with the grey matter cerebellum as the reference region. The time stability of mean regional SUVR was assessed for several regions for all subjects (Fig. 4) for Aβ and tau PET scans which showed that the values became stable for scan windows of ~ 30–50 min p.i. for [^18^F]AV45 and ~ 80–100 min p.i. for [^18^F]AV1451.

To investigate the relationship between the dynamic and static measures, the coefficient of determination (*R*^2^) was calculated for all atlas regions across all subjects between BP_ND_ values, obtained from SRTM and reference Logan graphical analysis methods, and SUVR values of several 20 min time windows. The relationship between these two outcome measures started to stabilize from scan windows of ~ 40–60 min p.i. for [^18^F]AV45 and ~ 80–100 min p.i. for [^18^F]AV1451. The linear regression analysis of the regional BP_ND_ and SUVR values for the above mentioned time windows showed a high correlation between the two measures using both dynamic methods for both Aβ (SUVR_40 − 60_ = 0.97 × BP_ND_SRTM_ − 1.2, *R*^2^_SRTM_ = 0.75; SUVR_40 − 60_ = 1.11 × BP_ND_Logan_ − 1.3, *R*^2^_Logan_ = 0.7; *p* < 0.01) and tau (SUVR_80 − 100_ = 1.028 × BP_ND_SRTM_ − 1.13; *R*^2^_SRTM_ = 0.88; SUVR_80 − 100_ = 1.27 × BP_ND_Logan_ − 1.25; *R*^2^_Logan_ = 0.71; *p* < 0.01) PET scans.

### Correlation between Aβ and tau tracer signals

**Fig. 5:**
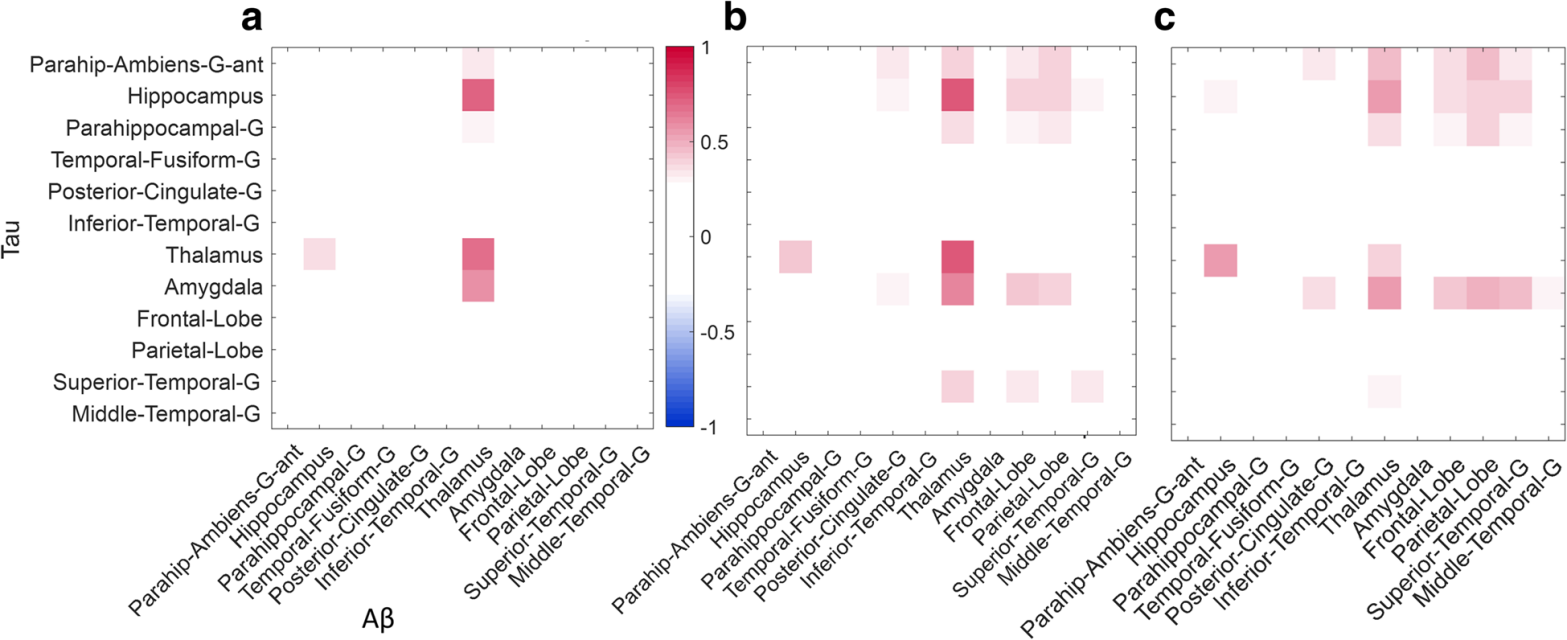
Signed coefficient of determination (*R*^2^) of regional BP_ND_ values derived using SRTM (**a**), reference Logan graphical analysis (**b**) and SUVR (**c**) methods for 12 regions with high potential for Aβ or tau uptake (The values between − 0.3 and 0.3 are masked for easier interpretation)

**Fig. 6:**
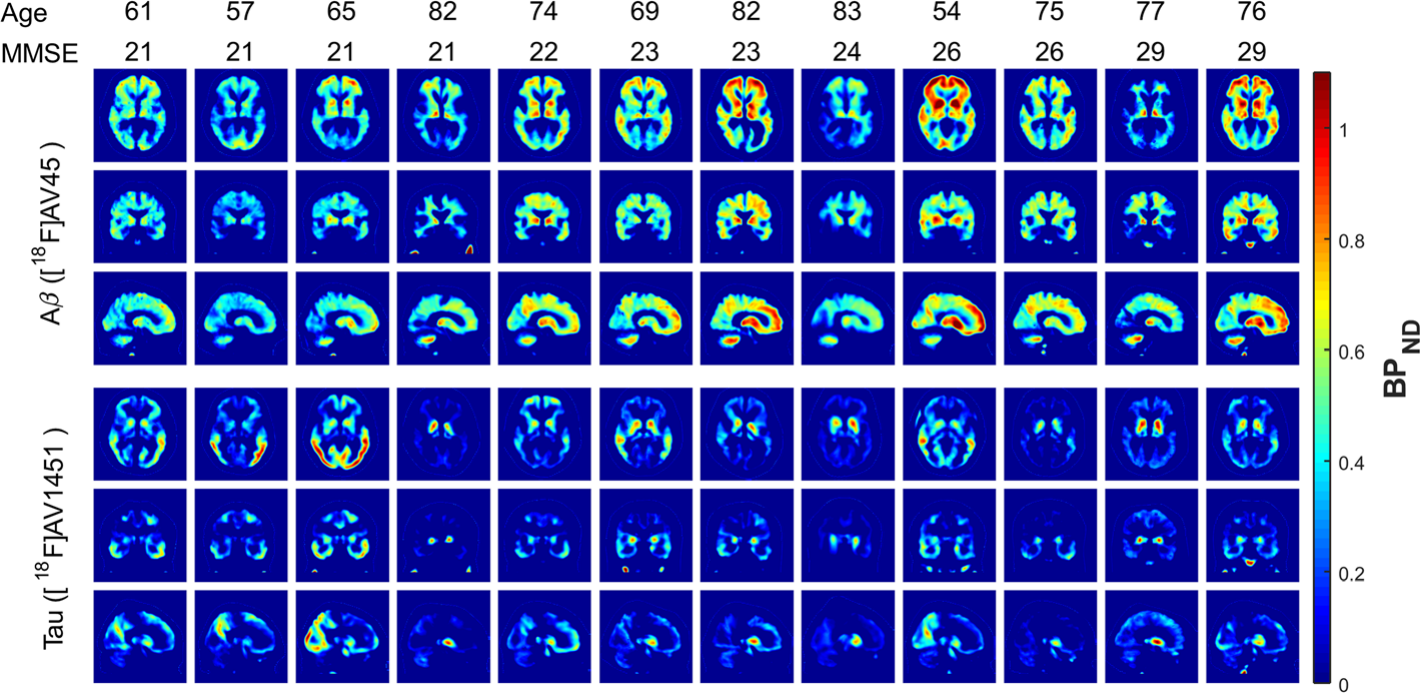
[^18^F]AV45 (top row) and [^18^F]AV1451 (bottom row) uptake patterns in whole brain represented by BP_ND_ values estimated using SRTM for all subjects who completed full 60 min of Aβ and at least 110 min of tau PET scan. Subjects are ordered based on their MMSE scores

The correlation between estimated regional Aβ and tau tracer signals using each of SRTM, reference Logan graphical analysis and SUVR was explored. Twelve different atlas regions with potential of having high Aβ and tau uptake were selected based on the literature and *R*^2^ was calculated for each outcome measure (BP_ND_ or SUVR) for all selected region pairs (Fig. 5). In particular, for the quantitative dynamic analysis methods (SRTM and reference Logan), Aβ signal in the thalamus was highly correlated with tau signal in thalamus (*R*^2^_SRTM_ = 0.76, *R*^2^_RefLogan_ = 0.84, *p* < 0.05), hippocampus (*R*^2^_SRTM_ = 0.74, *R*^2^_RefLogan_ = 0.73, *p* < 0.05) and amygdala (*R*^2^_SRTM_ = 0.56, *R*^2^_RefLogan_ = 0.55, *p* < 0.05). For SUVR, whilst the overall pattern of correlations was similar, *R*^2^ was reduced (hippocampus: *R*^2^_SUVR_ = 0.58, thalamus: *R*^2^_SUVR_ = 0.46 and amygdala: *R*^2^_SUVR_ = 0.57). The tracer uptake pattern in whole brain, for both [^18^F]AV45 and [^18^F]AV1451, are shown in Fig. 6 for all 12 subjects who completed full 60 min of the Aβ PET scan and at least 110 min of the tau PET scan. Overall, the tau signal was very low in all subjects but there was a strong signal observed in the striatal areas. Amyloid signal on the other hand was strong in all brain regions and was not directly related to MMSE scores (*R*^2^ = 0.006).

## Discussion

In this paper, we assessed the dynamic behaviour of [^18^F]AV45 and [^18^F]AV1451 targeting Aβ and tau proteins respectively in early AD subjects by estimating regional BP_ND_ values from dynamic PET scans using SRTM and reference Logan graphical analysis models with cerebellum grey as the reference region.

For the data presented here, arterial input functions were not available and therefore we considered SRTM results from full dynamic data as the reference standard based on previous studies [29, 30]. Comparison of the two dynamic analysis methods (SRTM and reference Logan graphical analysis) showed that they produced similar results with reference Logan graphical analysis slightly underestimating the BP_ND_ values.

Time stability analysis of both tracers demonstrated that stable estimates of the dynamic acquisition outcome parameter BP_ND_ could be obtained with a minimum acquisition time of 30 min for [^18^F]AV45 and 80 min for [^18^F]AV1451. Time stability analysis of the SUVR ratio for static acquisition scenarios indicated that the ratio became reasonably constant in scanning windows of 30–50 min p.i. for [^18^F]AV45 and 80–100 min p.i. for [^18^F]AV1451 and were well correlated with the full dynamic SRTM results across regions and subjects (*R*^2^ = 0.74 and *R*^2^ = 0.88, respectively) [31, 32]. These results are consistent with the faster kinetics of [^18^F]AV45 as compared to [^18^F]AV1451.

These results demonstrate that short static acquisitions, whilst reducing subject time in the scanner and improving compliance and feasibility, can derive semi-quantitative outcome measures that can be used for classification of subjects. Their use in longitudinal and intervention studies requires a little more caution as SUVR is not completely independent of blood flow changes [33].

Given the regional binding values for both tracers ([^18^F]AV45 and [^18^F]AV1451) in all subjects using SRTM, it was possible to explore the correlations between [^18^F]AV45 and [^18^F]AV1451 uptake in different brain regions. In this pilot data set, there was a high correlation between [^18^F]AV45 uptake in thalamus and [^18^F]AV1451 uptake in hippocampus. A degree of caution should be taken in interpreting these results given the low level of amyloid in the thalamus and issues with [^18^F]AV1451 due to off-target binding and possible spillover from the choroid plexus into the hippocampus. Nevertheless, from a biological stand point, the neuropathology literature [34] shows that dual amyloid and tau changes in the thalamus mirror those in the hippocampus. In addition, the changes in the thalamus affect preferentially those nuclei with hippocampal connections. Therefore, the current results, while speculative and potentially accounted for by off-target binding, suggest that linked thalamus-hippocampus dual pathology reported in neuropathology studies may be demonstrable using PET scans. Thus, it would be important to replicate these results with an improved tau tracer and in a larger cohort of subjects.

Whilst, [^18^F]AV45 and other EMA- and FDA-approved radiotracers have been shown to be highly selective for amyloid, the first generation of tau tracers, including [^18^F]AV1451, have demonstrated some off-target binding. Much of the off-target binding of putative tau agents has focused on monoamine oxidase (MAO) with [^18^F]AV1451 demonstrating some clear subcortical signals due to its binding to MAO-A [35, 36]. Thus, the interpretation of any subcortical [^18^F]AV1451 signal should be treated with a degree of caution. Other first generation tau tracers, such as [^18^F]THK5351 ([^18^F]GE216) [37, 38], have shown even more substantial problems with a large amount of off-target binding to MAO-B that also compromises the interpretation of cortical regions [35]. Second generation tau tracers are now just appearing with [^18^F]GTP1 [39], [^18^F]RO6958948 [40] and [^18^F]MK6240 [41] all demonstrating cleaner signals with increased selectivity for tau.

## Conclusions

Based on the data presented, short (20 min) static PET scans at appropriate time windows provide SUVR values which are in reasonable agreement with BP_ND_ values calculated from dynamic scans using SRTM. Appropriate scan window choices for [^18^F]AV45 are 30–50 or 40–60 min p.i., and for [^18^F]AV1451 are 80–100 or 90–110 min p.i. based on the desired accuracy and logistics. Care should be taken to determine whether this outcome measure is optimal in interventional studies.

## Abbreviations

AD: Alzheimer’s disease
ADAS-Cog: Alzheimer’s disease assessment scale-cognitive
Aβ: Amyloid-β
BET: Brain extraction tool
BP_ND_: Non-displaceable binding potential
CSF: Cerebrospinal fluid
CT: Computed tomography
DPUK: Dementias platform UK
EEG: Electroencephalography
EMA: European medicines agency
FBP: Filtered back projection
FDA: Food and drug agency
FMRIB: Functional MRI of the brain
FSL: FMRIB software library
GCP: Good clinical practice
HIS: Hachinski ischemic score
ICF: Informed consent form
ICH: International conference on harmonisation
MAO: Monoamine oxidase
MEG: Magnetoencephalography
MIAKAT: Molecular imaging and kinetic analysis toolbox
MMSE: Mini mental state examination
MNI: Montreal neurological institute
MRC: Medical research council
MRI: Magnetic resonance imaging
NFT: Neurofibrillary tangles
NIA-AA: National institute of aging-Alzheimer’s association
NIHR: National institute for health research
PET: Positron emission tomography
p.i.: post injection
ROI: Region of interest
SPM: Statistical parametric mapping
SRTM: Simplified reference tissue model
SUVR: Standardized uptake value ratio
TAC: Time activity curve
